# Glycosphingolipid–Protein Interaction in Signal Transduction

**DOI:** 10.3390/ijms17101732

**Published:** 2016-10-15

**Authors:** Domenico Russo, Seetharaman Parashuraman, Giovanni D’Angelo

**Affiliations:** 1 Institute of Protein Biochemistry National Research Council, Via P. Castellino 111, Naples 80131, Italy; r.parashuraman@ibp.cnr.it (S.P.); g.dangelo@ibp.cnr.it (G.D.); 2 Istituto di Ricovero e Cura a Carattere Scientifico SDN, Via Emanuele Gianturco 113, Naples 80143, Italy

**Keywords:** glycosphingolipid, signalling, glycan–protein interaction

## Abstract

Glycosphingolipids (GSLs) are a class of ceramide-based glycolipids essential for embryo development in mammals. The synthesis of specific GSLs depends on the expression of distinctive sets of GSL synthesizing enzymes that is tightly regulated during development. Several reports have described how cell surface receptors can be kept in a resting state or activate alternative signalling events as a consequence of their interaction with GSLs. Specific GSLs, indeed, interface with specific protein domains that are found in signalling molecules and which act as GSL sensors to modify signalling responses. The regulation exerted by GSLs on signal transduction is orthogonal to the ligand–receptor axis, as it usually does not directly interfere with the ligand binding to receptors. Due to their properties of adjustable production and orthogonal action on receptors, GSLs add a new dimension to the control of the signalling in development. GSLs can, indeed, dynamically influence progenitor cell response to morphogenetic stimuli, resulting in alternative differentiation fates. Here, we review the available literature on GSL–protein interactions and their effects on cell signalling and development.

## 1. Introduction

Glycosphingolipids (GSLs) are a heterogeneous class of membrane lipids that are constituted by complex glycan moieties linked by a glycosidic bond to a ceramide lipophilic backbone [1]. GSLs have aroused a special interest in the light of their peculiar structures. On the one hand, due to the biophysical properties of their hydrophobic portion, GSLs promote the formation of membrane nanoscopic domains. These domains regulate lateral partitioning of receptors and consequently their activation and recruitment of downstream components of signalling cascades [2,3,4,5,6,7,8,9,10,11,12,13]. The biophysical properties of GSLs are also important outside the membrane context with simple GSLs contributing to the maintenance of the integrity of the epidermis and of its barrier function [14,15,16]. On the other hand, GSLs have a surprisingly high number of different glycans constituting their headgroups. Although the biological and evolutionary meaning of this extensive variability is still largely unclear [17], the glycans on GSLs are in a favourable position to interact with the lumenal portions of membrane proteins and receptors [18]. Notably, indeed, over the last decades, a number of studies have demonstrated how GSLs influence the behaviour and signalling capability of membrane proteins [2,19,20,21,22,23,24,25,26,27,28,29].

Interestingly, specific GSL headgroups serve as receptors for viruses and microbial virulence factors [30,31,32,33,34,35,36,37,38,39,40,41,42,43,44,45,46,47,48,49,50,51,52,53,54,55,56,57,58,59,60,61,62,63,64,65,66,67,68,69,70,71,72,73,74,75,76,77,78,79,80,81,82,83,84,85,86,87,88] (Table 1), indicating that glycans in GSLs can be recognized with remarkable specificity by dedicated microbial proteins. In fact, a number of studies have demonstrated that GSL-glycan moieties establish interactions with endogenous proteins or glycans located on the plasma membrane of the same or of neighbouring cells. The specificity of these interactions depends on the precise composition of GSL-glycan sugar residues [20,23,29,89,90,91]. By this virtue, the glycan portion of the GSLs has the potential to interface with specific plasma membrane proteins to modify their activity [2,19,20,21,22,23,24,25,26,27,28,29] by carbohydrate–carbohydrate or protein–carbohydrate interactions [2,29,92].

While the mechanistic details of these GSL–protein interactions are often poorly understood, in a small number of cases, the existence of GSL-sensing domains (GSDs) in proteins has been demonstrated [6,29,93,94]. Nonetheless, the basic principles by which GSL-glycans are specifically perceived by GSDs are unknown. Even in the absence of this information, the available data suggest that GSLs constitute a regulatory layer acting orthogonally to the ligand–receptor–transducers module, which allows the dynamic fine-tuning of intracellular signalling. This is of particular interest in cell differentiation events. Indeed, precursor cells for given differentiation lineages might respond differently to morphogenetic stimuli as a consequence of exposing different GSLs on their cell surfaces. As a matter of fact, the GSL-dependent regulation is central for developmental processes as failure to synthesize specific GSLs results in developmental disorders in humans and in tissue specific phenotypes in model organisms [95,96,97,98,99,100,101].

In this review, we intend to discuss the role of GSLs as plastic regulators of signal transduction. To this aim, we review some examples of GSL–protein interactions, and discuss their molecular aspects, their impact on the regulation of cell signalling, along with their pathophysiological significance.

## 2. GSL Synthesis and Turnover

**Figure 1:**
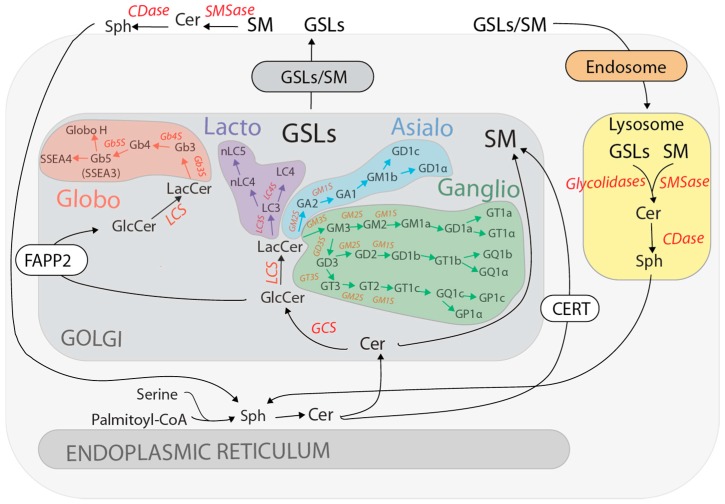
GSL synthesis and turnover. Schematic representation of the metabolism of the major GSL classes synthesized along the secretory and endocytic pathways. For details refer to the text. GSEs catalysing the major synthetic reactions and the enzymes involved in GSLs/SM dismantling are reported in red. GSLs, glycosphingolipids; Sph, sphinganine from the de novo pathway or sphingosine from the salvage pathway; Cer, ceramide; SM, sphingomyelin; GCS, GlcCer synthase; LCS, LacCer synthase; CERT, ceramide transfer protein; FAPP2, four-phosphate adaptor protein 2; SMSase, sphingomyelinase; CDase, ceramidase.

GSL metabolism is accomplished along the endomembrane system [1] (Figure 1). GSL synthesis starts in the endoplasmic reticulum (ER) where a sphingoid base is condensed with an acyl-CoA, to generate Ceramide (Cer) [102,103] (Figure 1). In the ER, Cer can be galactosylated to produce galactosylceramide (GalCer) [104]. GalCer is, then, transported to the Golgi complex where it can be sulphated to produce sulphogalactolipids, or sialylated to produce the GM4 ganglioside [1]. Alternatively, Cer can be conveyed to the Golgi complex via transport vesicles or by the ceramide-transfer protein CERT [105]. CERT transports Cer to the trans-Golgi network (TGN) where it is primarily used for the synthesis of sphingomyelin (SM) [105] (Figure 1). On the contrary, Cer transported to Golgi by vesicular transport is converted to glucosylceramide (GlcCer). GlcCer is synthesized by the addition of a glucose residue to Cer on the cytosolic leaflet of *cis*-Golgi membranes [105,106] and GlcCer is further glycosylated by the activity of glycosyltransferases distributed along Golgi *cisternae*, resulting in more than 300 species of GSLs. These enzymes transfer a specific carbohydrate from the appropriate activated sugar nucleotide (e.g., UDP-Galactose, CMP-sialic acid, UDP-Fucose, etc.) to the non-reducing end of the growing carbohydrate chain linked to GlcCer [107].

Similarly to Cer, GlcCer can be transported through two distinct routes. GlcCer is either transported across the Golgi complex via vesicular trafficking, or, alternatively, it is directly transferred by the lipid-transfer protein FAPP2 to the TGN [108,109,110,111] (Figure 1). Irrespective on its mode of transport, GlcCer is translocated to the luminal Golgi/TGN leaflet and undergoes a galactose (Gal) addition and conversion to lactosylceramide (LacCer). Once produced, LacCer cannot be translocated back to the cytosolic leaflet and constitutes the branching point for the synthesis of different GSL metabolic series [1]. These are defined by their internal core carbohydrate sequence [112,113] as ganglio (Gal-(Neu5Acα2-3)-β1-4glc) [114], globo (galα1-4gal) [115], lacto (galβ1-3glcNAcβ1-3gal) [116], and asialo (galNAcβ1-4gal) GSL series [117,118] (Figure 1). The synthesis of specific GSLs downstream of LacCer depends on multiple factors. The expression of a specific subset of glycosyltransferases, their subcellular localization, and the formation of multi-enzyme complexes all contribute to define the final GSL outcome. Moreover, substrate availability influenced by the action of lipid transfer proteins and sugar nucleotide transporters is another key parameter in this process [7,107,119,120].

Once at the TGN, newly synthesized GSLs and SM are transported by vesicular carriers to the plasma membrane. Here they can be further modified by plasma membrane located glycosidases, indicating that dynamic regulation of GSL composition can also occur at the cell surface [121]. From the plasma membrane, GSLs are internalized to the endosomal/lysosomal system. In the lysosomes a dedicated set of specific glycosylhydrolases, accessory proteins and acid ceramidases degrade GSLs to less complex compounds (glucose, Gal, hexosamine, sialic acid, ceramide, sphingosine, fatty acids) that are metabolically recycled for biosynthetic purposes (*salvage pathways*) [121,122] (Figure 1). A number of genetically derived human metabolic disorders result from defects in the lysosomal enzymes involved in GSL degradation and are commonly referred to as “GSL storage disorders” [8]. Importantly, the substrates of the defective enzymes accumulate in lysosomes and in many cases, the inability to degrade these compounds, induces a metabolic imbalance that leads to the secondary accumulation of GSLs [123]. For instance, increased levels of GM2 and GM3 gangliosides have been reported in patients affected by Niemann–Pick’s disease, where the primary storage material is SM [124]. A significant role in the pathogenesis of these diseases is attributed to the effects of the accumulated GSLs on cellular signalling pathways [123], highlighting the pathological consequences of aberrant GSLs composition on signalling.

## 3. GSL-Dependent Regulation of Signal Transduction

**Figure 2:**
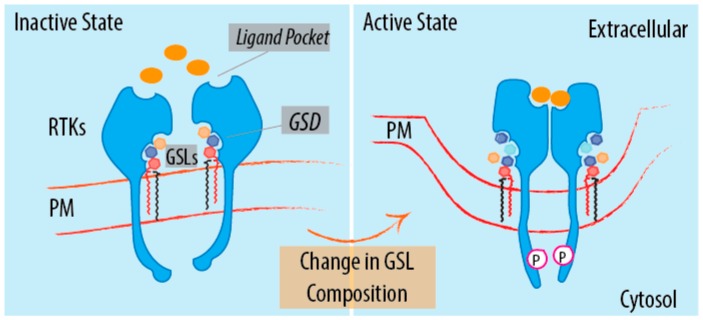
GSL influence on signalling. Schematic representation of the regulatory role of the GSLs on signal transduction. The regulation of RTKs by GSLs is provided as an example to illustrate the signalling role of GSLs. A specific GSL profile on plasma membrane can keep a RTK in an inactive state (**left panel**); A change in GSLs composition can regulate receptor activation/inactivation. GSLs act as allosteric regulators of receptor capability to recognize the ligand, to undergo multimerization and to auto-phosphorylate (**right panel**). GSLs, glycosphingolipids; Orange spheres represent ligands; Colored hexagons represent the different sugar residues of GSL-glycan moieties; Black-Red parallelogram, Cer backbone. Pink circle surrounding P represent phosphate groups; GSD, glycosphingolipid sensing domain; PM, plasma membrane; RTK, receptor tyrosine kinase.

Vertebrates possess a vast repertoire of GSLs, which differ according to the sugar composition, the anomeric linkages of the glycosidic bonds, and to the extent of branching of their glycans [1]. Since such complexity represents an exponential leap from the nearest evolutionary neighbour [125], it is tempting to speculate that this led to the emergence of a new level of regulation in vertebrate cells. A number of studies have indeed shown that GSLs regulate cellular signalling pathways by interacting with components of the signal transduction machinery (i.e., hormones, receptors and, intracellular transducers). These interactions have different outcomes: (i) allosteric regulation of the protein conformation; (ii) regulation of protein multimerization; (iii) protein segregation to membrane domains; and (iv) clustering of signalling molecules in proximity to their effectors [2,3,4,5,6,7,8,9,10,11,12,13] (Figure 2).

One paradigmatic example is that of the epidermal growth factor receptor (EGFR) [2,126,127]. EGFR was reported to be activated/inactivated depending on the GSLs composition of the membrane in which it resides. Epidermal Growth Factor (EGF) upon binding to EGFR stimulates the transition of the receptor from the inactive monomeric to an active homodimeric form. This event triggers the intrinsic receptor-associated tyrosine kinase activity, leading to receptor auto-phosphorylation [2,126,127] and to the activation of a signalling cascade that promotes cell proliferation. In 1986, Bremer et al. reported that EGFR auto-phosphorylation is inhibited by the exogenous addition of the GM3 ganglioside [126]. More recent studies approached the molecular details of this effect by in vitro studies using the purified receptor reconstituted in liposomes [2]. In spite of the absence of a known GSD, EGFR was reported to establish two different lateral interactions with GM3, having functional implications on the behaviour of the receptor. One is a protein–carbohydrate interaction involving the terminal *N*-acetylneuraminic acid of the GM3 and a lysine (Lys-642) localized in proximity to the transmembrane domain of EGFR. Through this interaction GM3 maintains the EGFR in its resting state preventing receptor dimerization and activation in absence of the ligand [2]. GM3 interacts with the EGFR also by a carbohydrate–carbohydrate interaction involving the sialylated Gal of GM3 and the terminal *N*-acetylglucosamine residues on EGFR *N*-glycans. In addition, this interaction, although weaker, was demonstrated to inhibit EGFR auto-phosphorylation and activation [128].

Apart from GM3, other gangliosides sharing the same glycan core structure as GM3 (i.e., GM1, GD1a and GT1b) have inhibitory effects on EGFR signalling [2,129]. On the contrary, the neutral GSL Gb4 exerts the opposite effect on EGFR [20]. Gb4, indeed, interacts directly with EGFR, potentiates its auto-phosphorylation capability and activates the phosphorylation of the downstream cascade components, ERK1/2. Of note, other neutral GSLs (i.e., LacCer and Gb3) do not have the same effect [20], indicating that GSL-mediated regulation depends on specific glycan configurations. Similar results were reported showing that gangliosides regulate different receptor tyrosine kinases like the fibroblast growth factor receptor [130,131,132], neurotrophin receptors [24,133,134], hepatocyte growth factor receptor [135,136,137], platelet-derived growth factor receptor [23,138,139], vascular endothelial growth factor receptor [140,141], and insulin receptor [27,142,143] (Figure 2). In some of these instances, it has been proposed that mono-sialylated gangliosides act as negative regulators while poly-sialylated gangliosides would activate the RTK signalling activity. Conflicting data have, nevertheless, challenged this interpretation suggesting that the regulatory function of gangliosides is not simply attributable to their sialic acid content [144]. Among these GSL–protein interactions, two are of special interest for their physiological importance: that of GM1 with neural tropomyosin receptor kinase A (TrkA), and that of GM3 with insulin receptor.

Nerve growth factor similarly to EGF activates its specific receptors (i.e., TrkA) by inducing dimerization and autophosphorylation. Already in the 1990s, seminal studies reported that the ganglioside GM1 specifically interacts with TrkA [24] to promote its dimerization [145] and consequent activation [24,133]. While the mechanistic aspects of GM1 interaction with TrkA are unknown, the interaction requires the glycosylation of the receptor [146] and its clustering with GM1 in membrane microdomains [147]. The neurotrophic effect of GM1 has been confirmed in neurodegenerative disease models like in Huntigton disease mice where GM1 metabolism was compromised and GM1 administration ameliorated the disease symptomatology [148,149]. Of clinical relevance is also the interaction of GM3 with the insulin receptor [27,150]. Here an inhibitory effect of GM3 on insulin signalling has been reported both in cell systems [27] and in mice where depletion on the GM3 synthesizing enzyme leads to increased insulin sensitivity [98]. Following on this experimental evidence, studies aimed at evaluating the possible association between GM3 ganglioside and diabetes/metabolic syndrome in humans have observed that high circulating levels of GM3 represent a risk factor for the development of insulin resistance [151,152].

A different example of how GSL-glycans regulate receptor function is the regulation of the Fas (CD95) receptor by Gb3 globoside. Here, Fas was found to bear a GSD that interacts specifically with Gb3 and LacCer but not with Gb4 or gangliosides [29]. The Fas-GSL interaction has important functional consequences as the GSD of Fas defines its internalization route, as well as the signalling outcome upon activation by the ligand. When paired to Gb3, the ligand-bound Fas is internalized by clathrin-dependent endocytosis. This results in the transduction of a cell death signal deriving from caspase-8 cascade activation. The disruption of the lipid–receptor interaction induces Fas to be internalized by ezrin-mediated endocytosis and to activate a pro-growth signalling through MAPK cascade signalling [29]. 

Another example of GSL regulation is that of mammalian Notch ligand Delta-like 1 (Dll1), where the GSD is present in the ligand itself [93]. The Notch signalling relies on a complex network where activation is regulated by both the signal-emitting and signal-receiving cell. Thus, first endocytosis and recycling of Dll1 are required to produce the activated form of the ligand and to enrich its surface level on the signal-emitting cells. Then, upon ligand–receptor interaction and endocytosis of this complex, a proteolytic cleavage of the receptor occurs causing the activation of the kinase signalling in the signal-receiving cells [93]. In co-culture assays, mutations in the key residues of the Dll1-GSD result in the rapid inactivation of Dll1 by degradation and inability to activate Notch signalling. Consistent with this finding, the authors also showed that inhibition of GSL production in the signal-emitting cells resulted in impaired Notch activation [9,93]. Thus, GSL–Dll1 interaction is required for a proper Notch signalling function. Indeed GSLs might either act as a docking platform to concentrate Dll1 to membrane microdomains specifically devoted to the Notch signalling, or increase Dll1 affinity for the Notch receptor [9,93].

Further complexity to the GSL-dependent signalling regulation is added by the heterogeneity in the ceramide backbones to which glycan moieties are bound [153,154]. With this respect many studies have underlined the involvement of cholesterol and GSL-hydrophobic portion in the formation of ordered membrane nanodomains, which can drive the clustering and distribution of receptors and non-receptor proteins (as reviewed in [155]). The GSL-ceramide backbone is also responsible of the direct interaction with transducer molecules. The physical linkage between GSL-ceramide backbone and some signalling mediators might be due to their post-translational fatty acylation, or to the presence of lipophilic protein domains able to mediate interaction with GSL-hydrophobic portion (as reviewed in [156,157]).

## 4. GSL-Sensing Domains (GSDs) as Sensors of GSLs

One of the first GSD to be discovered was that in the V3 loop of the gp120 glycoprotein of Human Immunodeficency Virus 1 (HIV-1) [158]. HIV uses the GSLs as alternative receptors to infect cells that do not express the canonical receptor CD4 [159]. Several GSLs are, indeed, recognized by gp120, including GalCer, 3′-sulphogalactosylceramide (SGC), GM3 ganglioside and Gb3 globoside [63,64,65,68,160]. The GSL binding site in gp12, consists of the amino-acidic sequence XXXGPGRAFXXX [161], which has been exploited as a molecular template for the discovery of endogenous proteins containing similar GSDs. Interestingly, gp120-type sequences have been identified in soluble proteins (i.e., synucleins and galectins [6,162]) as well as in transmembrane receptors. A gp120-type GSD is, indeed, present in the extracellular portion of the TNFα receptors super-family. For instance, the GSD molecular organization of the CD95 receptor consists of a hairpin structure containing two aromatic residues (Phe-133 and Phe-134) at the turn that strongly interact with LacCer and Gb3 but weakly with GD3 and Gb4. On the other hand, SM that lacks a sugar headgroup does not show any interaction [29]. A similar GSD has been found in the serotonin 1a receptor extracellular domain, with the LNKWTLGQVTC motif conserved in the whole serotonin receptor family. Interestingly, this specific sequence contains all the GSD hallmarks with basic (Lys-101), aromatic (Trp-102), and turn-inducing residues (Gly-105) [163].

**Figure 3:**
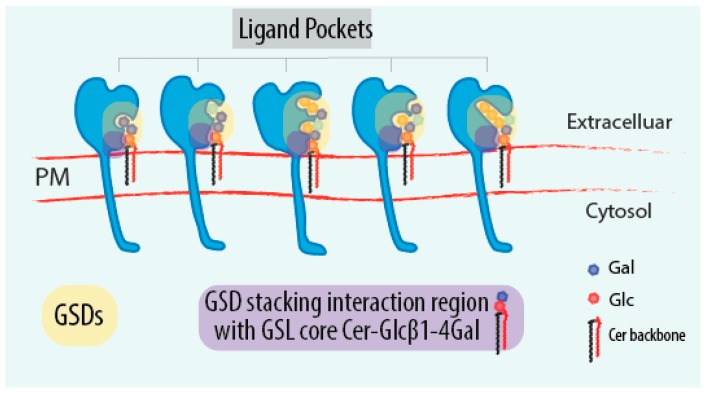
GSDs as sensors of the GSL-glycocode. Schematic representation of receptors containing different GSDs that recognize specificGSL-glycan portions. In light orange are highlighted GSDs in the receptors. In violet are indicated the conserved GSD portions recognizing the basic GSL core motif Cer-Glcβ1-4Gal present in the most GSLs. Coloured hexagons represent the different sugar residues of GSL-glycan moieties. Blue hexagon, Gal residue; Red hexagon, Glc residue; Black-Red parallelogram, Cer backbone; GSD, glycosphingolipid sensing domain; PM, plasma membrane.

The gp120-type GSDs, indeed, share common structural features. The gp120 GSD motif is nested within two α-helices and the central phenylalanine residue mediates the docking to a specific sugar ring in the GSL glycan portion [158]. Similarly gp120-type GSDs consist of a hairpin structure exposing an aromatic residue to the solvent, which plays a prominent role in protein-sugar interaction [29,163]. Carbohydrate-aromatic interactions usually occur at a number of axial CH groups located on the same face of the furanoses and/or pyranoses cyclic structure forming a planar apolar surface and the Pi electron density of the planar aromatic ring (CH:Pi stacking interactions). Replacing any of these aromatic residues with alanine results in decreased binding affinity [164]. The key sugar residue showing highest affinity to the aromatic amino acids is the Gal of the basic GSL core Cer-Glcβ1-4Gal motif present in most GSLs [164] (Figure 3). Nonetheless, the GSD of the gp120 V3 loop binds with greater affinity to Gb3 than GM3 ganglioside (both the GSL share the same “Cer-Glcβ1-4Gal” core), suggesting that molecular bonds not restricted to the common GSL core structure participate in the interaction (Figure 3).

In addition, some bacteria are able to exert cytotoxicity by using GSLs as a receptor for the entry of bacterial toxins into cells (Table 1). One of the best-characterized GSL-toxin interactions is that of Shiga toxin B subunit (ShTxB) with the Gb3 globoside [82]. As for the gp120-type of GSL–GSD interactions, in this case, the stacking interaction also involves aromatic residues and the Gal residues of Gb3 [165]. ShTxB is composed of five identical monomers, each of which has three Gb3-binding sites not all equally important for the binding. The first and third Gb3-binding sites are mainly involved in the interaction with the receptor [166]. Indeed, both these binding sites have key aromatic amino acids, Phe-30 in the first case, and the Trp-34 in the second, involved in the establishment of stacking interactions with the central and the terminal Gal residues of Gb3 respectively [165]. In addition, the ShTxB-GSD was used as a template to identify ShTx-type GSDs in endogenous proteins. Verotoxin B subunit (VTB), a Shiga-like toxin, was used for comparison in a multiple sequence alignment with the corresponding sequences in the extracellular domain of CD19 B cells receptor. When the CD19 primary sequence was aligned with a consensus sequence obtained from the VT1, VT2, and VT2e B-subunits, it showed overall 50% of identity. Moreover, high percentages of identity with CD19 were found in the region of the toxin where the Gb3-binding domain is located [167]. Interestingly the Glu-16, and Asp-17 residues present in the GSDs of VTB-subunits and potentially able to establish multiple hydrogen bonds with polar residue of the Gb3-trisaccharide were conserved also in the primary sequence of the CD19 receptor at the position 30 and 32 respectively. In addition, the aromatic residues Phe-30 and Trp-34 present in all the three VTB-subunits (as in ShTxB) and involved in the hydrophobic stacking interactions, are conserved in the CD19 receptor at the position 122 and 124 [167]. These considerations suggest the existence of a GSD in the CD19 extracellular domain and indicate a possible regulatory function for Gb3 on CD19 function. Nevertheless, the 3D structure of CD19 extracellular domain has not been solved and thus the existence of a GSD is not proven.

An interesting notion deriving from the reported data is that the Gal residue in the GSL core structure is involved in a default interaction with GSDs, while specificity is dictated by the surrounding sugar configuration (Figure 3). As we reviewed earlier [168], Gal is the most frequently found sugar in GSL glycans. Gal is also the most represented residue at even positions with other residues frequently found at odd positions when considering the occurrence of different residues along the reducing/non-reducing synthetic axis. Interestingly, when we measured the amount of the theoretical information content associated with the GSL glycan chain, we found that to anti-correlate with the position occupied by Gal with odd-positioned residues being extremely more information-rich than even-positioned ones. This makes us speculate that Gal residues are involved in the establishment of default interactions with GSDs while the other intercalating sugar residues determine binding specificity.

## 5. GSL Regulation in Development

Individual cells can survive and grow in the complete absence of GSLs while mammals require GSLs to complete their embryonic development [95,112,169], suggesting that GSLs play fundamental roles in multicellularity and development [18]. Accordingly the GSL composition of cells changes during differentiation, as a direct consequence of a change in the expression of specific GSL synthesizing enzymes [18,170,171,172]. The transcriptional programmes responsible for these changes are unknown, nonetheless GSLs are directly involved in the regulation of the differentiation processes. Thus knock out animal models for given GSLs synthesizing enzymes display specific developmental defects [168].

A prototypical case is that of neurogenesis, where a switch in GSL synthesis from globo and lacto series GSLs (synthesized by stem cells), toward the production of ganglio series GSLs at the stage of neuronal progenitor cells has been reported [18]. A further metabolic shift has been described in the transition from neuronal progenitor cells, that produce simple gangliosides such as GM3 and GD3, to mature neurons or glial cells showing an increased synthesis of more complex gangliosides such as GM1a, GD1a, GD1b and GT1b [173]. The role of GSLs in neural differentiation has been demonstrated by manipulating the GSL composition in various neuronal cell lines in culture and in animal models. According to these studies, GM3 and GD3 in NPCs contribute to β1-integrin expression to promote cell proliferation and self-renewal [174]. This creates a niche of precursors constantly sustaining adult neurogenesis [173]. Conversely, GM1 and GT1b promote neuronal differentiation and dendrite generation. GM1 and GT1b, indeed, by enhancing NGF-induced dimerization of TrkA and its phosphorylation promote the entry of neuronal progenitor cells into a postmitotic stage, and thus neuronal maturation [173]. Interestingly GM1, probably by this signalling, induces an epigenetic remodelling at level of the GM2/GD2 synthase promoter, increasing GM2/GD2 synthase expression and GM2 production. GM2 is then, directly converted into GM1 by GM1 synthase [175]. By this circuit GM1 establishes a positive feedback loop to promote neuronal maturation and sustain its own synthesis and that of GM1-derived gangliosides in the brain.

**Figure 4:**
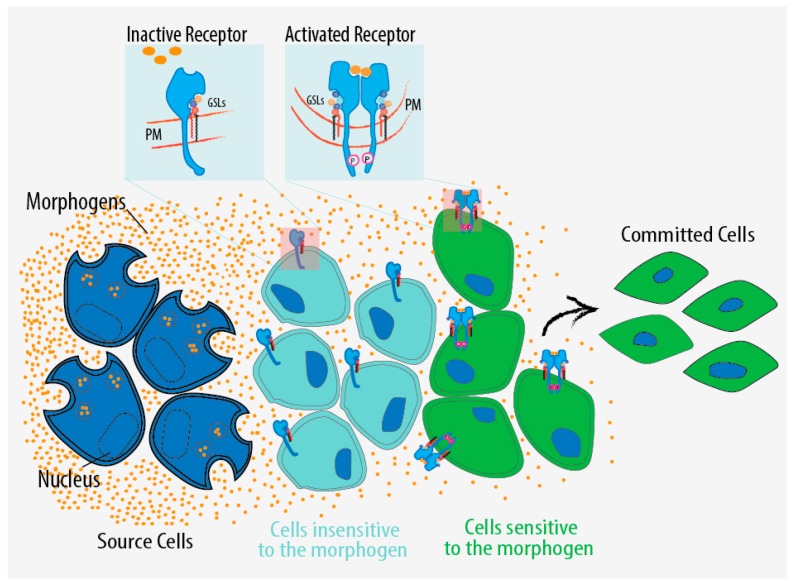
GSL regulation of signalling in development. Schematic representation of GSL-dependent regulation affecting the environmental sensing of the cell and the commitment to alternative differentiation fates. This regulatory layer might act in parallel to known mechanisms of tissue patterning such as morphogen gradients that induce the differentiation of specific cell types in a distinct spatial order. The same differentiation factor might be invisible to some cells and at the same time perceived by others depending on the fact that the target receptor is in a dormant or active state in relation to the GSL composition on the plasma membrane. Orange spheres represent morphogens; PM, plasma membrane.

These data suggest that brain-enriched gangliosides, probably by their regulatory function on signalling, modulate neuronal function and contribute to neuronal development by influencing the epigenetic state of the cell. By this mechanism gangliosides like GM1 have the potential to promote the expression of differentiation genes and thus to favour cell commitment to specific differentiation fates. This regulatory layer adds to known mechanisms of tissue patterning such as morphogen gradients that induce the differentiation of specific cell types in a distinct spatial order [176] (Figure 4). In this respect, GSL composition might determine the sensitivity of groups of cells, not necessarily located in a specific embryonic district to several morphogens. By these qualities, GSLs would provide the cells with the capability to stably maintain or modify their identity. On the other hand, the GSL dependent regulatory mechanism should also confer plasticity allowing cells to respond to sudden changes in the environment by remodelling their GSL composition without affecting the expression of the receptor pools on the plasma membrane (Figure 4).

Besides, the regulation of other enzymes of the sphingolipid metabolism like for example the ceramide synthases influence the GSL composition by the production of ceramides of different chain lengths [177]. Indeed, these ceramides are afterward incorporated into GlcCer, the precursor of most GSLs. The mechanisms that regulate the action of the six different known ceramide synthases go from transcriptional and post-translational regulation to altering enzyme activity by dimerization [177].

## 6. Open Questions

This review focuses on GSL–protein interactions and on their possible biological outcomes by reporting some examples where they have been studied thoroughly. Nevertheless, the extent of GSL involvement in cell regulation is not yet fully addressed. This knowledge gap derives from the lack of technologies that has hampered investigators to easily assess quantitatively and qualitatively the cellular GSL composition in physiopathological conditions and to reveal protein–GSL interactions. Thus, although GSDs have been recognized to bind GSL-glycan portions with different affinities, the basic principles accounting for specificity of these interactions are still far from being understood. As a consequence, future developments in the field will probably derive from solving the following issues.

### 6.1. What Are the Molecular Rules Driving GSL Sensing?

Glycan sequences in GSLs can be seen as a biological “language”, used by cells to specify their identities in multicellular contexts. To date, the rules determining how the information contained in GSL “words” is read are not understood. GSDs might act as sensors for specific GSLs. GSDs physically interact with GSL sugar residues by protein–carbohydrate and carbohydrate–carbohydrate interactions to sense the changes in the GSL composition, and consequently modify the activity of the protein in which they are embedded [29,163]. An important contribution to clarify details of the GSLs-GSD interactions has been provided by the studies on viral/bacterial GSDs [158,165]. Nevertheless, more effort is required to systematically identify new GSDs in the whole proteome, and to learn about their biochemical features. Indeed, to date it is unknown how many GSD types exist, and how they are structured. Moreover, further bioinformatics and biochemical studies are required to understand the role of GSL-glycan moieties in GSL–protein interactions. Finally, structural and dynamics data on GSD/GSL complexes are required to understand how each sugar residue interfaces with the surrounding amino acid counterpart. An interesting tool to study protein–lipid interactions and its dynamics in the bilayer context is the electron paramagnetic resonance (EPR) spectroscopy. EPR measures the magnetic moment of unpaired electrons contained in spin-labels (usually nitroxyl groups) that are synthetically introduced in the lipid of interest [178]. Spin-labelled lipids show changes in their EPR spectra when in proximity of interacting proteins. These spectral changes can be exploited to infer the stoichiometry, strength and the time scale of a specific protein–lipid interaction [179]. While EPR has been widely used to study the bilayer properties influenced by GSLs [180,181] few studies have directly approached GSL–protein interaction by the use of EPR [182] thus leaving space for future investigation.

### 6.2. Which Are the Targets of GSL-Dependent Regulation?

GSLs exert their biological functions by binding to specific proteins and regulating their activities. The list of proteins interacting with specific GSLs is slowly but steadily growing and comprises a number of plasma membrane receptors as well as tetraspanins, integrins, and caveolins [18]. The functional outcomes of these interactions have been defined in some cases and include both activatory and repressive regulations. Nevertheless the number of reported GSL–protein interactions is limited. As a result, to date, we lack an estimation of the fraction of the proteome that interacts with specific GSLs and of protein domains/motifs involved in these interactions. GSL–protein interaction studies have suffered from the absence of methods to systematically approach this issue. Indeed, large-scale screenings based on the identification of GSL–protein interactions have yet not been performed. Probably, the most promising approach to tackle this issue is based on photochemistry. By using a photo-activable analogue of the GSLs, containing a photo-activable diazirine group, GSLs can be covalently crosslinked to neighbouring/interacting proteins by UV irradiation [183]. Recent developments of this technique have made available bi-functional sphingolipid analogues, where in addition to the photo-activable diazirine group a terminal alkyne moiety was included in the sphingosine structure that allows tagging of protein–lipid complexes by click chemistry [184]. Thus, by the cycloaddiction of a fluorophore-azide to the terminal alkine group of the bi-functional sphingolipid, it is now possible to visualize the lipid–protein crosslinked complexes. Alternatively, the cycloaddiction of a tag-azide group (e.g., biotin-azide) allows the immunoprecipitation and the identification by mass spectrometry of the lipid–protein crosslinked complexes. By the use of this technique it will be possible in a near future to systematically reveal GSL–protein interactions. The scale up of this approach will possibly lead to the construction of a map of the GSL-interactome, which might be used to understand the GSDs–GSL recognition code.

### 6.3. What Is the Role of the Hydrophobic GSL Portion in the Regulation of Signal Transduction?

While not discussed in this review the role of GSL hydrophobic portion in the signalling control is not of minor importance. Indeed, GSL-ceramides act as organizers of membrane domains by inducing heterogeneous membrane partitioning and specialization of functional membrane domains. In addition, here, there are still many aspects to be clarified regarding how protein binding specificity is conferred by parameters such as the length of the acyl chains of the ceramide, the degree of unsaturation and how these features can specifically modulate signal transduction. The combination of all these parameters yields a further level of complexity [154], which constitute an additional information reservoir being perceived by direct protein interactors, and involved in the GSL-dependent fine tuning of cell signalling.

### 6.4. How Is GSL Metabolism Regulated?

Since GSLs act on a regulatory level integrating with the classical ligand–receptor module, aberrations in GSL composition result in signalling defects, which can be corrected by appropriately manipulating the GSL metabolism. This requires a deep knowledge on the spatiotemporal regulation of the GSL synthesis, which is influenced by multiple parameters. Importantly, the transcriptional programs regulating the expression of specific GSL glycosyltransferases, their physical interactions and sub-Golgi compartmentalization, as well as the accessibility to their substrates are parameters that we still have not completely unravelled. Thus, although the GSL metabolism has been satisfactorily described, we have little clue of how GSL synthesis is controlled. Future studies focused on the regulation of GSL metabolism, should provide a more systematic view on the GSL role in signalling and organism pathophysiology.

### 6.5. Which Is the Role of the GSL-Dependent Regulation in Development?

Once bound to their interacting partners, GSLs modulate their activities. By the virtue of this property, GSLs impact on cell signalling and ultimately on cellular transcriptional programmes. Nevertheless a comprehensive picture of the impact of GSL composition on cell signalling and transcriptional regulation is lacking. Moreover, although GSLs are widely recognized regulators of developmental processes, only a modest number of GSLs have been studied in any real detail, thus leaving the understanding of the specific roles of most GSLs in driving differentiation processes to future research.

## Figures and Tables

**Table 1:** Glycosphingolipids (GSLs) headgroups serve as receptors for viruses and bacterial toxins.

**Virus**	**Glycosphingolipid Receptors**	**References**
Picornaviridae		
Porcine sapelovirus (PSV)	GD1a	[30]
Caliciviridae		
Human Norovirus (HuNoV)	Type 1, 2, 3 HBGA	[31,32]
Human Norovirus (HuNoV): GII.4 strain	H, B, and A type 1 Lewis b	[33]
Murine Norovirus (MNV): MNV-1 and CR3 strains	GD1a; GT1b	[34,35]
Bovine Norovirus (BoNoV)	HBGA	[36]
Rabbit Hemorrhagic Disease Virus (RHDV)	A and H Type 2 HBGA	[37]
Adenoviridae		
Adenovirus type 37 (Ad37)	GD1a	[38]
Reoviridae		
Reovirus serotype 1 (T1)	GM2	[39]
Porcine Rotavirus: OSU strain	GM3	[40,41]
Porcine Rotavirus: CRW-8 strain	GD1a	[42]
Porcine Rotavirus: TFR-41 strain	Unknown ganglioside	[43]
Simian Rotavirus: SA11 strain	NeuGcGM3, IV^3^NeuAcLc4, GM2, GD1a	[44]
Simian Rhesus Rotavirus: RRV strain	Unknown ganglioside	[43,45]
Bovine Rotavirus: NCVD strain	NeuGcGM3, IV^3^NeuAcLc4, GM2, GD1a	[44,46]
Bovine Rotavirus: UK strain	NeuGcGM3, GM1, GD1a, GM2, IV^3^NeuAcLc4	[43,46]
Human Rotavirus: KU, MO, DS-1 and Wa strains	GM3, GM1	[42,43,47,48,49]
Polyomaviridae		
Trichodysplasia spinulosa-associated Polyomavirus (TSPyV)	GM1	[50]
Murine Polyomavirus (MPyV)	GD1a, GT1b	[51,52]
Simian Virus 40 (SV40)	GM1	[44,53,54]
BK Virus (BKV)	GD1b, GT1b	[55]
JC Virus (JCV)	GT1b	[56]
Merkel Cell Polyomavirus (MCPyV)	GT1b	[57]
Parvoviridae		
Human Parvovirus B19	Gb4, SSEA-3, SSEA-4, nLc4	[58,59]
Simian Parvovirus	Gb4; Forssmann antigen	[60]
Bovine Adeno-associated Virus (BAAV)	Unknown ganglioside	[61]
Retroviridae		
Human Immunodeficiency Virus (HIV)	Gb3, GM3, GalCer, GD3, SM4 sulfatide	[62,63,64,65,66,67,68,69]
Flaviviridae		
Dengue virus (DENV) type 2	GM3, nLc4	[70,71,72]
Orthomyxoviridae		
Influenza A virus, subtype H3N2: A/Victoria/3/75 strain	Ganglioside with Neu5Acα2-3Galβ1-4 (Fucα1-3) GlcNAc epitope; nLc8, nLc10 and nLc12	[73]
Influenza A virus, subtype H3N2: A/Hiroshima/52/2005 strain		
Poxviridae		
Vaccinia virus (VACV): Western-Reserve strain	SM4 sulfatide	[74,75]
Paramyxoviridae		
ParamyxoVirus 1 (Newcastle Disease)	GM3, GM2, GM1, GD1a	[76]
Sendai virus (SV) (murine parainfluenza virus type 1)	GD1a, GQ1b, IV^3^NeuAcLc4, nLC4	[77,78]
Human parainfluenza virus types 1 (Hpiv-1)	IV3NeuAcLc4, Nlc4	[78]
Human parainfluenza virus types 3 (hPIV-3)		
**Bacterial Toxin**	**Glycosphingolipid Receptors**	**References**
Cholera toxin Vibrio cholera	GM1	[79,80]
Heat labile toxin 1 Escherichia coli	GM1	[81]
Shiga Toxin Shighella dysenteriae	Gb3	[82,83]
Shiga-like toxins (SLT1 and SLT 2) *Escherichia coli* (Verotoxins)	Gb4	[84,85]
Tetanus neurotoxin (TeNT) *Clostridium tetani*	GT1b, GD1b	[86]
Botulinium toxin BoNT *Clostridium botulinum*	GT1b, GD1a	[87]
Heat labile toxin IIB *Escherichia coli*	GD1a	[88]
